# Supplementary material for: No severe genetic bottleneck in a rapidly range-expanding bumblebee pollinator
Source: Proc Biol Sci. 2021 Feb 10;288(1944):20202639. doi: 10.1098/rspb.2020.2639 (PMC7893223; doi:10.1098/rspb.2020.2639)
Supplement: Electronic supplementary material for 'No severe genetic bottleneck in a rapidly range-expanding bumblebee' [file rspb20202639supp1.pdf]

Electronic supplementary material for:

No severe genetic bottleneck in a rapidly range-expanding bumblebee pollinator (*Proceedings of the Royal Society B*, DOI: 10.1098/rspb.2020.2639)

Ryan E. Brock [1, †, *], Liam P. Crowther [1, †], David J. Wright [1, 2], David S. Richardson [1], Claire Carvell [3], Martin I. Taylor [1] & Andrew F. G. Bourke [1, *]

[1] School of Biological Sciences, University of East Anglia, Norwich Research Park, Norwich, Norfolk NR4 7TJ, UK

[2] Earlham Institute, Norwich Research Park, Colney Lane, Norwich, Norfolk NR4 7UZ, UK

[3] UK Centre for Ecology & Hydrology, Maclean Building, Crowmarsh Gifford, Wallingford, Oxfordshire OX10 8BB, UK

[*] Corresponding authors: ryan.brock@uea.ac.uk; a.bourke@uea.ac.uk

[†] These authors contributed equally to the study

1. Supplementary methods

(a) Genetic diversity and bottleneck analysis

(i) Worker sample collection and genotyping

Workers were sampled from a *Bombus hypnorum* population in Norwich, Norfolk, UK, with *B. hypnorum* having been first recorded in the sampling area in 2008 [1]. A total of 675 *B. hypnorum* workers were sampled from a 2 × 2 km area of suburban Norwich from 15 May 2014 to 16 June 2014 (n = 398) and from 28 May 2015 to 1 July 2015 (n = 277), these being the same samples as those from which mating frequency, worker foraging distance, nest density, between-year lineage survival rate, and isolation by distance were reported by Crowther *et al.* [2]. Free-flying *B. hypnorum* workers were captured with a handheld insect net, and tissue for DNA extraction was non-lethally sampled by clipping off the tarsal tip of a mid-leg [3]. Each tarsal tip was then stored in 100% ethanol at room temperature until DNA extraction.

DNA was extracted from the tarsal tips of all workers using an ammonium-acetate ethanol precipitation procedure modified from Richardson *et al.* [4]. Prior to tissue digestion, tarsal tips were frozen with liquid nitrogen for two minutes before being ground into powder. To maximise DNA yield, all samples underwent ethanol precipitation at -20°C for at least three hours. Extracted DNA was suspended in low-T.E. buffer (10 mM Tris.HCl, 0.1mM EDTA) and the quality of extractions was checked using a Nanodrop 8000 (Thermo Fisher Scientific, UK).

All PCRs were carried out following the procedure outlined in Crowther *et al.* [2], with all samples being genotyped, in three primer multiplexes, across the 14 microsatellite loci characterised in that study. These microsatellite loci had previously been shown to meet the following expectations across the two sampling years in the Norwich population: 1) all loci

were in Hardy-Weinberg equilibrium; 2) no null alleles; and 3) no loci showed evidence of linkage disequilibrium [2]. Each PCR plate included: (1) a negative control, consisting of all reagents and primers but no template DNA; and (2) a positive control in the form of DNA extracted from two *B. hypnorum* queens whose genotypes were known from previous characterisation.

PCR products were visualised with a 48-well capillary ABI 3730 DNA analyser and a ROX-500 internal size standard (Thermo Fisher Scientific, UK), and peaks were determined using GeneMapper 4.0 software (Thermo Fisher Scientific, UK). Alleles were accepted only if they appeared in two or more workers.

From regenotyping samples, the per-locus mean (range) error rate from mistyping was 2.26% (0.91% - 3.17%) [2]. The negative controls did not show any peaks corresponding to the amplified alleles. A total of 645 workers (2014: n = 375; 2015: n = 270) were genotyped at up to 14 loci (median [range] = 11 [6-14] loci) [2].

(ii) Genetic diversity and bottleneck evaluation

To avoid pseudoreplication by sampling multiple related workers, the bottleneck analyses used only one randomly sampled worker from each cluster inferred using COLONY v2 [5], with each cluster consisting of the matrilineal descendants of a single unsampled 2014 colony queen. Clusters consisted of all workers that were related as full or half-sisters, allowing within-year relatedness under queen polyandry to be accounted for, or all workers that were related as aunts and nieces, allowing between-year relatedness to be accounted for. This way, 89 distinct clusters were identified among all workers sampled across 2014 and 2015 (n = 645), thus producing 89 unrelated worker genotypes for the bottleneck analysis.

Two different methods were used to test for a bottleneck in the *B. hypnorum* study population. First, a sign test was implemented using the program BOTTLENECK 1.2.02 [6].

Given that recently bottlenecked populations are expected to show a faster reduction in allele number than in heterozygosity, the sign test can determine the extent of a bottleneck by testing for an excess of expected heterozygosity over that expected under mutation-drift equilibrium [6,7]. The sign test analysis assumed a two-phase model of allelic mutation and a 9:1 ratio of one-step to multi-step mutations, as these assumptions have been shown to be most applicable to microsatellite loci [8].

Second, a test for a bottleneck was conducted by calculating the M-ratio [9] across each locus for the *B. hypnorum* study population. The M-ratio defines the ratio between the number of alleles at a locus (k) and the size range in base pairs (r) of those alleles. Reductions in population size reduce k (by causing alleles to be lost at random through sampling effects). However, since losing only the smallest or largest alleles will reduce r, a reduction in k will occur faster than a reduction in r, and so the ratio of k to r will decrease. Hence, bottlenecked populations should show lower M-ratios than non-bottlenecked populations. Under the same assumptions as for the bottleneck sign test, an M-ratio of less than 0.7 was taken to indicate evidence of a historical population reduction [9].

A reduced M-ratio could occur from either colonisation of the UK arising from a small founding population or historical population reductions unlinked to *B. hypnorum*'s colonisation of the UK. To address this, M-ratios were calculated for five other UK *Bombus* species (*B. hortorum*, *B. lapidarius*, *B. pascuorum*, *B. ruderatus*, and *B. terrestris*; henceforth described as 'reference' *Bombus* species) using microsatellite genotyping data collected from single populations of workers sampled in 2011 at a site in southern England by Dreier *et al.* [10]. The dataset of Dreier *et al.* [10] was selected for comparison with the *B. hypnorum* data because of its taxonomic breadth, relative geographic proximity of the sampling site to Norfolk, similar number of loci used for genotyping (range = 10–14), overlap of molecular markers used between studies (percent range shared microsatellite loci = 21.0–57.1%), and a

worker sampling protocol similar to that used in the current study. Importantly, populations of the reference species have not undergone range expansions within the UK, and four of the species (all except the scarce *B. ruderatus*) are common and widespread. Hence, populations of these species should, at most, exhibit evidence of genetic bottlenecks associated with population fluctuations expected for long-established UK native *Bombus* species. M-ratios calculated for the reference *Bombus* species therefore provided a null model against which the M-ratio calculated for the range-expanding *B. hypnorum* was compared, with the expectation that if *B. hypnorum* had suffered a severe genetic bottleneck upon colonising the UK, it would exhibit a lower M-ratio in comparison to the reference *Bombus* species.

(b) Diploid male production and allelic diversity at the sex-determining locus

(i) Male sample collection and genotyping

B. hypnorum males were sampled from two sources. First, 20 mature *B. hypnorum* colonies were collected from the field in Norfolk and Suffolk, UK, from 15 May to 25 June 2017 (n = 17), and from 31 May to 21 June 2018 (n = 3; Table S1). Nests were sought via appeals for them to apiculturists (via the Norfolk Beekeepers' Association) and to the general public (via the social media platforms Facebook and Twitter). Once collected, colonies were frozen at -20°C, and all individuals were censused according to developmental stage (i.e. egg, larvae, pupae, and adults), sex and, for females, caste (Table S1). To determine male genotypes from field-collected nests, only male pupae were genotyped, since it is possible that adult males found within these nests had entered them from different nests [11,12]. Male pupae were distinguished from female pupae by one of two traits: (1) the presence of externally visible 'claspers', the male reproductive organs formed of the sagittae, squama, stipes, volsella, and spatha, at the abdominal tip instead of a sting; and (2) the presence of 11 antennal

flagellomeres instead of 10 as in females (Figure S1). Male pupae were sampled for genotyping at random from colonies, with all 20 colonies providing at least seven male pupae and up to 24 male pupae being sampled from colonies that contained 25 or more male pupae. In total, 380 male pupae across the 20 colonies (2017: $n_{male\ pupae} = 337$; 2018: $n_{male\ pupae} = 43$) were sampled for genotyping (Table S1).

Second, adult males were sampled from *B. hypnorum* colonies reared in the laboratory from field-collected queens. Given that diploid male production (DMP) negatively impacts colony productivity in *Bombus* [13,14], genotyping males sampled from mature field nests alone might underestimate the frequency of diploid males (i.e. colonies exhibiting DMP might be less likely to grow large enough to be reported for collection by members of the public). Further, sampling free-flying, field-collected males to estimate DMP levels [e.g. 15–17] might also underestimate the frequency of DMP due to colony survivorship bias. Sampling adult males from field-collected queen-reared colonies is therefore likely to provide a more reliable estimate of levels of diploid male production and matched mating within a population. Adult rather than pupal males were sampled from the laboratory-reared colonies because these colonies were kept in separate nest-boxes with no possibility of mixing.

To rear colonies, 107 *B. hypnorum* queens were collected from Great Windsor Park, Surrey (n = 25), Chingford, Greater London (n = 7), and various sites across Norwich, Norfolk (n = 75) from 7 March to 19 April 2018 (Table S2). Queens were reared under constant conditions (mean ± range: 27°C ± 1°C; 60% ± 10% RH) and provided with pollen (Sussex Wholefoods, Eastbourne, UK) and 50% Apiinvert sugar syrup (Südzucker AG, Mannheim, Germany) *ad libitum*. Their colonies were checked daily for adult offspring production, with all adult workers produced being individually marked on eclosion with numbered discs (EH Thorne Ltd, Market Rasen, UK), and all adult sexuals (males and young queens) being removed on eclosion and frozen at -20°C. This was because, in nature, adult sexuals are known to disperse

from the nest a few days after eclosion [18]. Adult males were distinguished from adult workers by any one of three traits: (1) the presence of a blunt-ended abdomen tip, concealing the male reproductive apparatus; (2) the lack of a corbicula (pollen basket) on the tibia of each hind-leg; and (3) the presence of 11 antennal flagellomeres instead of 10 as in females (Figure S2).

Daily checks of adult offspring allowed the collation of colony demographic data and male production schedules for each successfully reared colony across its lifespan (defined for each colony as the time between first and last offspring eclosion; Table S3). In total, 37 of the 107 collected queens (34.6%) successfully reared at least one adult offspring (of which 11, 3, and 23 were from the Surrey, Greater London, and Norwich populations, respectively; Table S3). Of these 37 colonies, 9 produced only workers, 6 produced only males, and 22 produced both workers and males (Figure S3; Table S3). Of the 22 colonies that produced both workers and males, six produced at least some males that eclosed within one week of first worker eclosion (mean [range] number of days between first worker eclosion and first male eclosion = 3 [0-5] days; Table S4), and 16 produced males that all eclosed more than one week after first worker eclosion (mean [range] number of days between first worker eclosion and first male eclosion = 31 [19-40] days; Table S5). Hence, of the 28 colonies that produced males, 12 were assigned as ‘first-brood male’ producers (i.e. the six colonies in which only males were produced, plus the six colonies in which some males eclosed within one week of first worker eclosion; mean [range] n of first-brood males produced per colony = 2 [1-7]), because in all cases adult males will have developed from the first batch of eggs laid by the queen (Table S4). Five of these colonies (89, 120, 133, 138 and 159) also went on to produce males that eclosed later than one week after first worker eclosion (Table S4). The remaining 16 colonies, in which all males had eclosed later than one week after first worker eclosion, were assigned as ‘late male’ producers, because these adult males will have developed from the second or

later batches of eggs laid by the queen (Table S5). Hence, the two classes of colony were exclusive in terms of timing of male production (males in first brood vs. no males in first brood), but five of the first-brood male producing colonies went on to produce late males as well (i.e. males were produced in both the first and subsequent broods).

For each male-producing colony, up to the first 10 adult males to eclose were selected for genotyping. However, for colonies producing first-brood males and more than 10 males across their entire colony lifespan (colonies 120, 138 and 159), or producing only late males but producing at least one diploid male in the initial 10 adult males selected for genotyping (colony 83), the sample size was increased, with the first 24 males to eclose being selected for genotyping (Tables S3, S4, S5). The sampling date of each individual sampled male was noted, and as all sampled males were sampled shortly after their eclosion (i.e. as callows), this allowed the sequence of eclosion for all genotyped males within a given colony to be known (Tables S4, S5). Using these methods, a total of 84 males were sampled from the first-brood male producing colonies, comprising 25 first-brood males (defined as those males that eclosed within one week of first worker eclosion or were produced instead of workers) and 59 late males (defined as those males that eclosed later than one week after first worker eclosion). Further, a total of 148 males (all of which were late males) were sampled from late male producing colonies. Hence, a total of 232 adult males from 28 field-collected queen-reared colonies were selected for genotyping, comprising 25 first-brood males (i.e. all available first-brood males produced by first-brood male producing colonies) and 207 late males (Table S3). All sampled males were kept at -20˚C until DNA extraction.

For all males (n = 380 pupal males from field-collected nests plus 232 adult males from colonies reared from field-collected queens = 612 males in total), DNA was extracted from the thoracic tissue of each male (pupal or adult) and PCRs were performed using the methods described for workers above [2]. Similarly, all male samples were genotyped at the same 14

microsatellite loci as those used for the workers. Each PCR plate included (1) a negative control, comprising all reagents and primers but no template DNA, and (2) a positive control comprising a single haploid male or a single diploid queen with known genotypes. Given that some colony queens produced only a single male, alleles were accepted if they appeared in one or more males, so that rare alleles in the population were not missed. The negative controls did not show any peaks corresponding to the amplified alleles. Marker BTMS0132 was later found to be monomorphic across all typed males and was therefore dropped from the analysis. The genotypes of 612 males used in analyses therefore came from up to 13 loci (median [range] = 13 [3-13] loci), with 67.5% of males being successfully genotyped at all 13 loci.

(ii) Estimation of levels of diploid male production

Males were considered diploid if they were phenotypic males (Figures S1, S2) that were heterozygous at two or more of the microsatellite loci. All males in colonies (both field-collected and those reared from field-collected queens) in which diploid males were detected (by this criterion) during the first round of genotyping (field-collected colonies: $n_{colonies} = 2$, $n_{males} = 48$; colonies reared from field-collected queens: $n_{colonies} = 6$, $n_{males} = 70$) underwent re-extraction of DNA and re-genotyping, to ensure that diploidy was not called on the basis of contamination. In two colonies, two males (56MP5 and 135M1), representing 0.33% of all genotyped males, appeared diploid during the first round of genotyping and haploid during the second round of genotyping. Therefore, these males underwent DNA extraction and genotyping for a third time, allowing them to be assigned as definitively haploid or diploid. Both males appeared homozygous across all loci in the second and third rounds of genotyping and were therefore classed as haploid. To ensure that haploidy of males sampled from first-brood male producing colonies was not called on the basis of large allele dropout, whereby alleles with longer sequence length are missed, all sampled males that appeared as

haploid from the first-brood male producing colonies ($n_{colonies}$ = 8, $n_{first\text{-}brood\ males}$ = 21, $n_{late\ males}$ = 19) also underwent re-extraction of DNA and re-genotyping.

These two regenotyping procedures, accounting for 25.8% of all sampled males, were also used to calculate locus-specific allele error rates from mistyping for the male genotyping. On this basis, the per-locus mean (range) allelic error rate was estimated to be 1.17% (0.00-7.25%) (Table S6). Allelic richness and allele frequency were calculated using Cervus v3.0.7.

Given that any diploid males are expected to show similar levels of heterozygosity to workers (i.e., in the present case, having at least three heterozygous loci, since the minimum number of these loci at which workers from the Norwich population [2] were heterozygous was three), the initial scoring of two heterozygous loci for diploidy may not have been conservative enough. Hence, to ensure that the number of loci at which males had to be heterozygous to be considered diploid did not affect our results, the diploid male classification was re-run. Here, two further criteria were introduced for diploid male classification, with males classified as diploid if they were heterozygous at: i) one or more loci (less conservative); or ii) three or more loci (more conservative).

Finally, the phenotypes of all males genetically confirmed as diploid (as described above) were double-checked (by inspection of the original individual pupal and adult males sampled) to confirm that they were not workers that had been mistakenly phenotyped as males (Figures S1, S2). Double-checking of phenotypes confirmed all individuals classified as diploid males by the above procedures were males, and not misidentified workers.

In summary, for a male to be assigned as diploid in the final data set, it was required to be scored as heterozygous across at least two microsatellite loci during at least two independent rounds of DNA extraction and genotyping, and also to have undergone double-checking to confirm its male phenotype.

In the 37 colonies reared from field-collected queens, genotyping results confirmed that five queens (76, 135, 141, 146, and 164) produced no workers and only haploid males (Table S3 & S4). These queens might have represented individuals that failed to mate before entering hibernation the previous year, the proportion of which can reach as high as 11% in certain *Bombus* populations [19], and were therefore incapable of producing diploid offspring upon colony foundation. Hence, to assess the mating status of these queens, their abdomens were dissected in Ringer solution under a Leica M80 stereomicroscope (Leica Microsystems, Milton Keynes, UK). The spermatheca was isolated from the rest of the reproductive system [2] and removed to check for the presence or absence of stored sperm under an Olympus BX41 (Olympus Life Sciences, Southend, UK) phase-contrast compound microscope (Figure S4).

The frequency of colonies exhibiting DMP was estimated from the diploid male data in three ways. The first used the frequency of colonies with DMP in the field-collected colonies producing diploid offspring. The second used the frequency of colonies with DMP in the colonies reared from field-collected queens that produced diploid offspring. The third allowed for potential sampling error in determining DMP in the colonies reared from field-collected queens that produced diploid offspring, as some of these colonies produced only small numbers of males for genotyping (Figure 2b, 2c). This third method included, from the colonies reared from field-collected queens that produced diploid offspring, only those colonies in which the sum of the numbers of workers and/or males produced was three or more. This was because the five colonies found to have produced diploid males (Figure 2b, 2c) all produced at least one diploid male among their first three males (Tables S4, S5). Hence, if a colony were producing diploid males, it would have been expected to produce at least one diploid male in its first three diploid offspring (including those for which the

fertilization event in fact led to their being sex-determined as workers), or in the first three male offspring that were then selected for genotyping.

(iii) Estimation of allelic diversity at the sex-determining locus

From Adams *et al*. [20], if N is the number of alleles at the sex-determining locus in a system of *sl*-CSD, and p is the per-mating frequency of matched mating (i.e. heterozygote queen mates with a hemizygous male sharing one of her sex-determination alleles), then, as a matched mating may happen in two ways (AB × A or AB × B), each (assuming equal allele frequencies) with probability $1/N$, $p = 2 \times (1/N)$, or:

$$N = 1 / (0.5p). \qquad \text{(Equation S1)}$$

In a system of monogyny (single queen per colony) and obligate single mating (monandry), p may be estimated from the frequency of colonies showing DMP (D) simply as $p = D$ (as each colony is headed by a single queen mated once [19,21]). However, under monogyny and facultative polyandry, this is not the case, since a single colony exhibiting DMP and headed by (for example) a doubly-mated queen might represent a queen with either one or two matched matings (the additional matched mating being 'masked' in the latter case). Monogyny is universal in temperate *Bombus* species, but, unlike many *Bombus* species, *B. hypnorum* exhibits facultatively polyandry [2]. In the main study UK *B. hypnorum* population, queens were estimated to mate 1, 2, or 3 times at a frequency of 34%, 59%, and 7%, respectively (yielding a mean queen mating frequency of 1.7) [2]. The following approach was therefore taken to estimate p from D:

Let colonies headed by a queen mating 1, 2, or 3 times occur in the proportions X, Y, and Z, respectively, and let the probability that each of these classes of colony contains a queen with at least one matched mating be P_1, P_2, and P_3, respectively. Then the frequency of colonies showing DMP is given by:

$$D = X(P_1) + Y(P_2) + Z(P_3). \quad \text{(Equation S2)}$$

$P_1 = p$ (as above) and, from the binomial theorem, $P_2 = [1 - (1 - p)^2]$ and $P_3 = [1 - (1 - p)^3]$. Therefore:

$$D = X[p] + Y[1 - (1 - p)^2] + Z[1 - (1 - p)^3]. \quad \text{(Equation S3)}$$

Rearranged, this yields:

$$ap^3 + bp^2 + cp + d = 0, \quad \text{(Equation S4)}$$

where: $a = Z$, $b = -Y - 3Z$, $c = X + 2Y + 3Z$, and $d = -D$. Given observed values for X, Y, Z, and D, this cubic equation can then be solved for p for the case when $0 < p \leq 1$. From the calculated value of p, Equation S1 then yields N.

For X, Y, and Z, the observed proportions of queens mating at each frequency in the main study population from [2] were used, i.e. 0.34, 0.59, and 0.07, respectively. Values of D came from the three different methods used to estimate the frequency of DMP within diploid-producing colonies as outlined in the previous section: (1) from the field-collected colonies; (2) from the colonies reared from field-collected queens; and (3) from the colonies reared from field-collected queens after correcting for sampling error in the sampling of male offspring for genotyping.

As the estimates of X, Y, and Z found in the study population by [2] might have been measured with error and/or could vary across years, and as other *B. hypnorum* populations might exhibit different levels of facultative polyandry, values of p (and hence N) were also calculated assuming, as boundary conditions, either (1) 100% single mating of queens (mean queen mating frequency = 1.0) or (2) 50% double mating and 50% triple mating of queens (mean queen mating frequency = 2.5), i.e. assuming either $X = 1$, $Y = 0$, $Z = 0$, or $X = 0$, $Y = 0.5$, $Z = 0.5$, respectively. For this, Equation S4 was used with D equal to the value estimated

from the colonies reared from field-collected queens after correcting for sampling error, as this was assumed to be the most accurate estimate of *D*.

2. Supplementary results

Diploid male production and allelic diversity at the sex-determining locus

(i) Levels of diploid male production

Diploid males were heterozygous at a mean (range) of 5.5 (2-9) loci. The total number of colonies exhibiting DMP remained the same regardless of the minimum number of heterozygous loci (one to three) used to assign diploid males (Table S7). However, changing the minimum number of heterozygous loci deemed to classify an individual male as diploid changed the total number of diploid males estimated, though this change involved a difference of one male only to the total number estimated and occurred via effects in a single colony (colony 49), with 17, 17, and 16 males from this colony being assigned as diploid according to whether the criterion for assignment was a minimum of one, two, or three heterozygous loci, respectively (Table S7). As the estimate of total number of colonies exhibiting DMP was unaffected, the identity of the colonies classified as DMP colonies was also unaffected, and the effect on the estimated total number of diploid males was very small (the totals being 50, 50, or 49 diploid males), the criterion of a minimum of two heterozygous loci to assign male diploidy was retained.

Frequency of DMP in field-collected colonies: One of the 20 field-collected colonies (5%), all of which produced diploid workers, exhibited DMP (Figure 2a; Table S1). Hence *D* was estimated to equal 1/20 = 0.05.

Diploid males accounted for 4.47% of all genotyped male pupae across the 20 colonies ($n_{haploid\ males}$ = 363, $n_{diploid\ males}$ = 17). In the single field-collected colony that exhibited DMP (colony 49), diploid males accounted for 70.8% of all sampled male pupae ($n_{haploid\ males}$ = 7, $n_{diploid\ males}$ = 17).

Given that 70.8% of the genotyped male pupae in colony 49 were diploid, 46 of the total 65 male pupae (Table S1) were estimated to be diploid ($65 \times 0.708 = 46.0$). Colony 49 also produced 74 diploid female pupae (73 worker-destined + 1 queen-destined pupae). Hence, a Mendelian ratio (diploid males / all diploid offspring) of 38.3% was estimated for the single field-collected colony that exhibited DMP ($46 / (46 + 74) = 0.383$). Note that a Mendelian ratio of 50% would be expected only in DMP colonies with single mating; in a DMP colony under polyandry, the ratio would depend on the number of mates of the queen, the proportion of matings that were matched, and relative usage of sperm from the different males by the queen.

Frequency of DMP in colonies reared from field-collected queens: Genotyping of adult males from the 2018 colonies reared from field-collected queens revealed that five of the 37 colonies produced no workers and only haploid males (mean number (range) of haploid males produced in such colonies = 2.2 (1-3)). Queen mating status was assessed by dissection of the spermatheca in three of the five colonies (76, 141, and 146) that produced only haploid males and no workers. Mating status of the remaining two queens (135 and 164) could not be assessed due to degradation of tissue inside the abdomen. All three queens in which mating status could be determined had stored sperm inside the spermatheca, indicating that they were mated (Figure S4). Therefore, unmatedness was not the cause of exclusively haploid male production within these colonies. Nonetheless, as these five colonies did not produce any diploid offspring, they were excluded from estimates of DMP frequency, and subsequently the calculation of allelic diversity at the sex-determining locus (see below).

In total, 37 of the 107 field-collected queens reared at least one adult offspring (Table S3), with nine colonies producing only workers, six colonies producing only males, and 22 colonies producing both workers and males. All 6 + 22 = 28 colonies producing males were sampled for diploid males (by genotyping). Of the six colonies producing only males, five

were found to produce haploid males only (and were excluded as above) and one (colony 102) to produce a single diploid male (Figure 2b, 2c; Table S3). Hence, 32 colonies produced diploid offspring (nine colonies that produced only workers, 22 colonies that produced workers and males, and the male-only producing colony that produced a single diploid male). Four of the 22 colonies producing workers and males produced diploid males (Figure 2b, 2c; Table S3). Overall, therefore, of the 32 colonies producing diploid offspring, five colonies (15.6%) exhibited DMP. Hence *D* was estimated to equal 5/32 = 0.156.

The five DMP colonies comprised four of the 12 (33.3%) first-brood male producing colonies and one of the 16 (6.3%) late-male producing colonies (Figure 2b, 2c; Table S3). The four first-brood male producing colonies that exhibited DMP produced one first-brood male each and in each case it proved to be a diploid male (Table S3, S4). Further, three of these colonies (colonies 133, 138, and 159) also went on to produce late males, with, in each colony, some of these males being diploid males (56.5–100% of late males) (Table S3, S4). In the eight first-brood male producing colonies that did not exhibit DMP (66.7% of all first-brood male producing colonies; Figure 2b), a mean (range) of 2.6 (1-7) first-brood males were produced, all of which were haploid (Table S3).

Overall, diploid males accounted for 14.2% of all sampled males across the 32 colonies ($n_{haploid\ males} = 199$, $n_{diploid\ males} = 33$). Across all five diploid male-producing colonies, diploid males represented 48.5% of all sampled males ($n_{haploid\ males} = 35$, $n_{diploid\ males} = 33$) and, within each colony, accounted for a range of 16.6-100% of the sampled males.

As above, Mendelian ratios (diploid males / all diploid offspring) were calculated for the five colonies exhibiting DMP for the period of time over which a colony was producing diploid offspring. Mendelian ratios of 22.2%, 100%, 50%, 29.2%, and 18.8% were calculated for

colonies 83, 102, 133, 138, and 159, respectively. Overall, the mean Mendelian ratio for those colonies that exhibited DMP was 44.0%.

Frequency of DMP in colonies reared from field-collected queens with correction for sampling error: Of the 32 colonies producing diploid offspring (as above), 26 produced a total of three or more workers and/or males (five of the nine colonies producing only workers, 21 of the 22 colonies producing workers and males, but not the male-only producing colony that produced a single diploid male) (Table S3). Of these 26 colonies, four colonies (15.4%) exhibited DMP. Hence D was estimated to equal 4/26 = 0.154.

(ii) Allelic diversity at the sex-determining locus

Solving Equation S4 for p given X, Y, and Z = 0.34, 0.59, and 0.07, respectively, and D = 0.05, 0.156, or 0.154 (from the above estimates), yielded p = 0.029, 0.094, or 0.093, respectively. Hence, from Equation S1, the number of alleles at the sex-determining locus was estimated in the three cases as N = 69.0, 21.3 or 21.5, respectively.

Using Equation S4 and applying the boundary conditions of either (1) 100% single mating of queens or (2) 50% double mating and 50% triple mating of queens, and taking D = 0.154 (value estimated from the colonies reared from field-collected queens after correcting for sampling error), returned values of p of 0.154 and 0.065, respectively. In turn, these yielded values of N of 13.0 and 30.8, respectively. From these calculations, increasing polyandry increased the estimate of N, because a given number of colonies represents a greater number of matings over which the frequency of matched matings is calculated, but did not do so proportionately (i.e. in this case by a factor of 2.5), because in rare cases matched matings are 'masked' by others in the polyandrous queens. Therefore, taking the best estimate of N as that calculated when D was estimated from the colonies reared from field-collected queens after

correcting for sampling error, i.e. $N = 21.5$, bounds for this value were estimated as 13.0 and 30.8.

References

1. BWARS. 2019 *Bombus hypnorum* mapping project. See https://www.bwars.com/content/bombus-hypnorum-mapping-project (accessed on 11 October 2019).

2. Crowther LP, Wright DJ, Richardson DS, Carvell C, Bourke AFG. 2019 Spatial ecology of a range-expanding bumble bee pollinator. *Ecol. Evol.* **9**, 1–12. (doi:10.1002/ece3.4722)

3. Holehouse KA, Hammond RL, Bourke AFG. 2003 Non-lethal sampling of DNA from bumble bees for conservation genetics. *Insectes Soc.* **50**, 277–285.

4. Richardson DS, Jury FL, Blaakmeer K, Komdeur J, Burke T. 2001 Parentage assignment and extra-group paternity in a cooperative breeder: the Seychelles warbler (*Acrocephalus sechellensis*). *Mol. Ecol.* **10**, 2263–2273.

5. Jones OR, Wang J. 2010 COLONY: a program for parentage and sibship inference from multilocus genotype data. *Mol. Ecol. Resour.* **10**, 551–555. (doi:10.1111/j.1755-0998.2009.02787.x)

6. Piry S, Luikart G, Cornuet JM. 1996 BOTTLENECK: A computer program for detecting recent reductions in the effective population size using allele frequency data. *J. Hered.* **90**, 502–503.

7. Cornuet JM, Luikart G. 1996 Description and power analysis of two tests for detecting recent population bottlenecks from allele frequency data. *Genetics* **144**, 2001–2014.

8. Di Rienzo A, Peterson AC, Garza JC, Valdes AM, Slatkin M, Freimer NB. 1994 Mutational processes of simple-sequence repeat loci in human populations. *Proc. Natl. Acad. Sci. United States Am.* **91**, 3166–3170.

9. Garza JC, Williamson EG. 2001 Detection of reduction in population size using data from microsatellite loci. *Mol. Ecol.* **10**, 305–318.

10. Dreier S, Redhead JW, Warren IA, Bourke AFG, Heard MS, Jordan WC, Sumner S, Wang J, Carvell C. 2014 Fine-scale spatial genetic structure of common and declining bumble bees across an agricultural landscape. *Mol. Ecol.* **23**, 3384–3395.

11. Lopez-Vaamonde C, Koning JW, Brown RM, Jordan WC, Bourke AFG. 2004 Social parasitism by male-producing reproductive workers in a eusocial insect. *Nature* **430**, 557–560.

12. Paxton RJ, Thorén PA, Estoup A, Tengö J. 2001 Queen-worker conflict over male production and the sex ratio in a facultatively polyandrous bumblebee, *Bombus hypnorum*: The consequences of nest usurpation. *Mol. Ecol.* **10**, 2489–2498. (doi:10.1046/j.0962-1083)

13. Gosterit A. 2016 Adverse effects of inbreeding on colony foundation success in bumblebees, *Bombus terrestris* (Hymenoptera: Apidae). *Appl. Entomol. Zool.* **51**, 521–526.

14. Whitehorn PR, Tinsley MC, Brown MJ, Darvill B, Goulson D. 2009 Impacts of inbreeding on bumblebee colony fitness under field conditions. *BMC Evol. Biol.* **9**, 152.

15. Darvill B, Ellis JS, Lye GC, Goulson D. 2006 Population structure and inbreeding in a rare and declining bumblebee, *Bombus muscorum* (Hymenoptera: Apidae). *Mol. Ecol.* **15**, 601–611.

16. Ellis JS, Knight ME, Darvill B, Goulson D. 2006 Extremely low effective population sizes, genetic structuring and reduced genetic diversity in a threatened bumblebee

species, *Bombus sylvarum* (Hymenoptera: Apidae). *Mol. Ecol.* **15**, 4375–4386.

17. Charman TG, Sears J, Green RE, Bourke AFG. 2010 Conservation genetics, foraging distance and nest density of the scarce Great Yellow Bumblebee (*Bombus distinguendus*). *Mol. Ecol.* **19**, 2661–2674.

18. Alford D V. 2011 *Bumblebees*. Hebden Bridge, UK: Northern Bee Books.

19. Takahashi J, Ayabe T, Mitsuhata M, Shimizu I, Ono M. 2008 Diploid male production in a rare and locally distributed bumblebee, *Bombus florilegus* (Hymenoptera, Apidae). *Insectes Soc.* **55**, 43–50.

20. Adams J, Rothman ED, Kerr WE, Paulino ZL. 1977 Estimation of the number of sex alleles and queen matings from diploid male frequencies in a population of *Apis mellifera*. *Genetics* **86**, 583–596.

21. Jones CM, Brown MJF. 2014 Parasites and genetic diversity in an invasive bumblebee. *J. Anim. Ecol.* **8**, 1428–1440. (doi:10.1111/1365-2656.12235)

22. Maebe K, Karise R, Meeus I, Mänd M, Smagghe G. 2019 Pattern of population structuring between Belgian and Estonian bumblebees. *Sci. Rep.* **9**, 1–8. (doi:10.1038/s41598-019-46188-7)

23. Maebe K, Meeus I, Vray S, Claeys T, Dekoninck W, Boevé J, Rasmont P, Smagghe G. 2016 A century of temporal stability of genetic diversity in wild bumblebees. *Sci. Rep.* **6**, 38289. (doi:10.1038/srep38289)

SUPPLEMENTARY FIGURES

Figure S1. The morphological features used to distinguish between pupal male (top: a – c) and female (bottom: d – f) *Bombus hypnorum*. See also [18]. (a) Head of a male pupa showing 11 antennal flagellomeres (labelled 1 – 11); (b) Ventral view of the abdominal tip of early-stage male pupa, showing the externally visible sagittae and squama that, along with the stipes, volsella, and spatha, make up the male reproductive apparatus; (c) Ventral view of the abdominal tip of late-stage male pupa, showing same features as in (b); (d) Head of a female pupa showing 10 antennal flagellomeres (labelled 1 – 10); (e) Ventral view of the abdominal tip of early-stage female pupa, showing the externally visible lancet and sheath lobes that make up the stinging apparatus; (f) Ventral view of the abdominal tip of late-stage female pupa, showing same features as in (e). Images taken using a GXCam HiChrome-S (GTVision Ltd, Stansfield, UK) attached to a Leica M80 stereomicroscope (Leica Microsystems, Milton Keynes, UK).

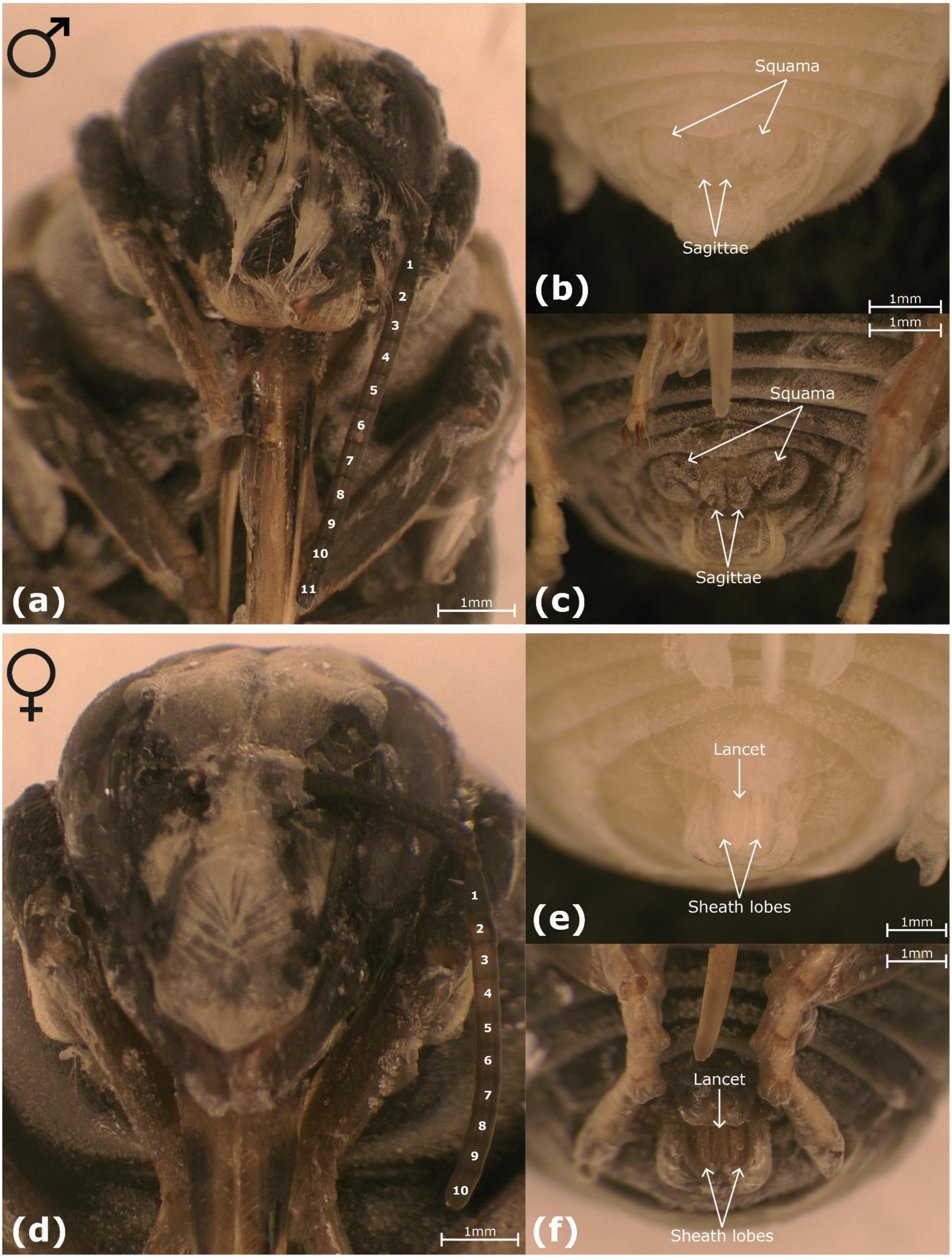
♂
1
2
3
4
5
6
7
8
9
10
11
(a)
1mm
Squama
Sagittae
(b)
1mm
1mm
Squama
Sagittae
(c)
♀
1
2
3
4
5
6
7
8
9
10
(d)
1mm
Lancet
Sheath lobes
(e)
1mm
1mm
Lancet
Sheath lobes
(f)

487

Figure S2. The morphological features used to distinguish between adult male (top: a – c) and female (bottom: d – f) *Bombus hypnorum*. See also Alford [18]. (a) Head of an adult male, showing 11 antennal flagellomeres (labelled 1 – 11); (b) Ventral view of the abdominal tip of an adult male, showing a blunt-ended abdomen tip that conceals the male reproductive apparatus; (c) Tibial hind-leg of an adult male, showing lack of a corbicula (pollen basket); (d) Head of an adult female, showing 10 antennal flagellomeres (labelled 1 – 10); (e) Ventral view of the abdominal tip of an adult female, showing a pointed ending that conceals the reproductive and stinging apparatus; (f) Tibial hind-leg of an adult female, showing presence of a corbicula. Images taken using a GXCam HiChrome-S (GTVision Ltd, Stansfield, UK) attached to a Leica M80 stereomicroscope (Leica Microsystems, Milton Keynes, UK).

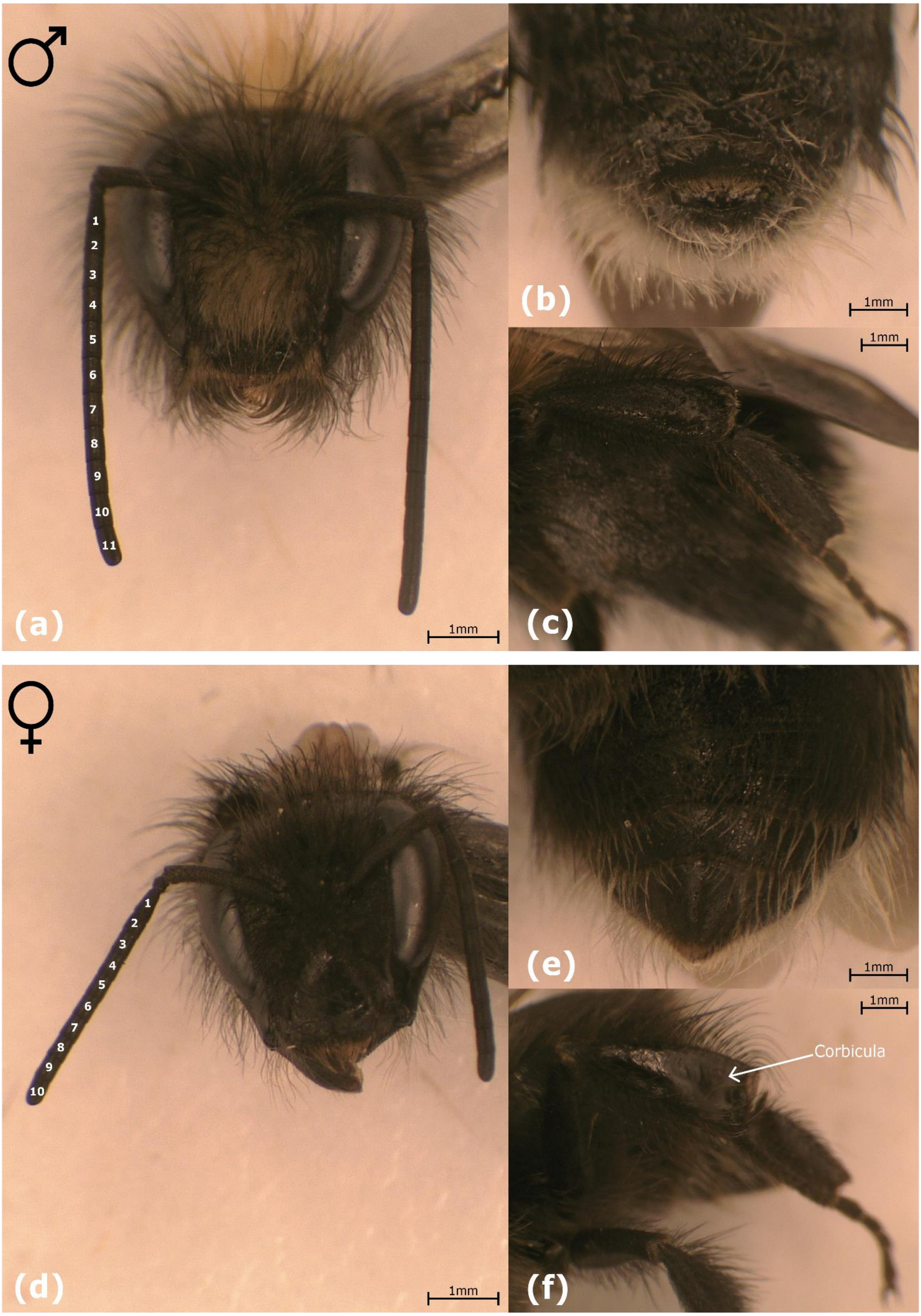
♂
1
2
3
4
5
6
7
8
9
10
11
(a)
1mm
(b)
1mm
1mm
(c)
♀
1
2
3
4
5
6
7
8
9
10
(d)
1mm
(e)
1mm
1mm
Corbicula
(f)

Figure S3. Outcomes of colonies reared from 2018 field-collected *Bombus hypnorum* queens in the current study. Of the 107 collected queens, 70 failed to rear any adult offspring, while 37 reared at least one adult offspring. Of these 37 colonies, 9 produced only workers, 6 produced only males, and 22 produced both workers and males. All 28 male-producing colonies were sampled for diploid male production (by genotyping of males) (Table S3). Of the 22 colonies that produced both workers and males, six produced ‘first-brood males’ (i.e. males that eclosed within one week of first worker production), and 16 produced ‘late males’ (i.e. males that eclosed a week or more after first worker eclosion). Hence, 12 first-brood male producing colonies (the six colonies that produced only males, plus the six that produced first-brood males and workers) and 16 late male producing colonies were defined. The two classes of colony were exclusive in terms of timing of male production (males in first brood vs. no males in first brood), but five of the first-brood male producing colonies went on to produce late males as well (i.e. males were produced in both the first and subsequent broods). Subsequent genotyping confirmed that one of the six male-only producing queens produced diploid males. Hence, there were 32 queens that produced diploid offspring and were subsequently used to estimate DMP frequency, matched mating frequency and the number of alleles at the sex-determining locus.

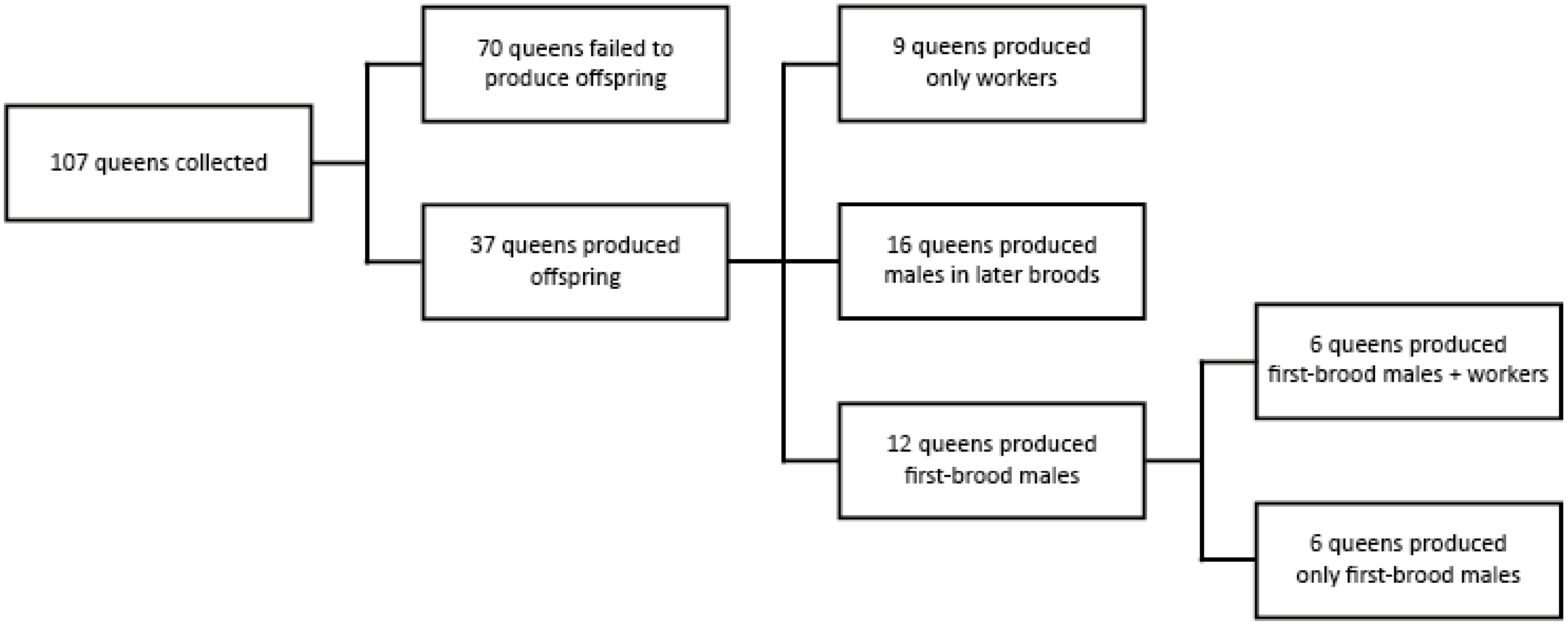
107 queens collected
70 queens failed to produce offspring
37 queens produced offspring
9 queens produced only workers
16 queens produced males in later broods
12 queens produced first-brood males
6 queens produced first-brood males + workers
6 queens produced only first-brood males

Figure S4. Spermathecae from *Bombus hypnorum* queens, dissected to check for mating status (see also Crowther *et al.* [2]): (a) Spermatheca from a mated queen, showing the sperm packet inside; (b) Spermatheca from an unmated queen, lacking a sperm packet. Spermatheca images taken using a GXCam HiChrome-Met (GTVision Ltd, Stansfield, UK) attached to an Olympus BX41 (Olympus Life Sciences, Southend, UK) phase-contrast compound microscope.

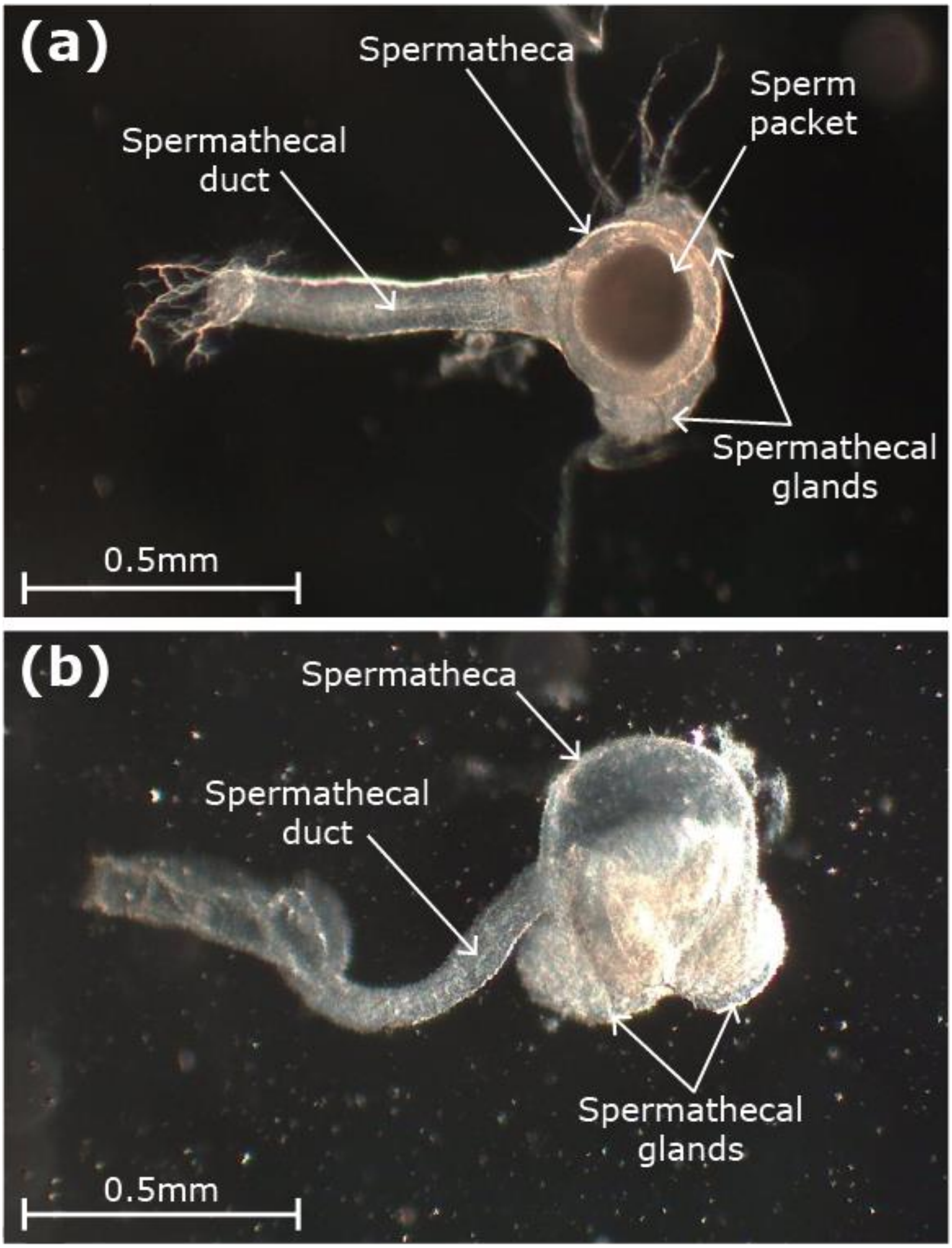
(a)
Spermatheca
Sperm
packet
Spermathecal
duct
Spermathecal
glands
0.5mm
(b)
Spermatheca
Spermathecal
duct
Spermathecal
glands
0.5mm

524

SUPPLEMENTARY TABLES

Table S1. Collection details and colony composition for 20 *Bombus hypnorum* nests collected in the field from sites in Norfolk and Suffolk, UK, from 15 May to 25 June 2017 and from 31 May to 21 June 2018. Colony ID: identification number of colony. Asterisk next to the colony ID number indicates the colony produced diploid males. Collection date: date colony collected from field. Collection location: site of colony collection. Collection co-ordinates: latitude and longitude of colony collection location. N adult queens, workers, males: numbers of adult queens, workers, and males, respectively, present at time of collection. N queen pupae, worker pupae, male pupae: numbers of queen, worker, and male pupae, respectively, present at time of collection. N genotyped male pupae: number of male pupae, of the total number present (in N male pupae column), randomly sampled for genotyping in the current study.

Colony ID	Collection date (dd/mm/yyyy)	Collection location	Collection co-ordinates (Lat, Long)	N adult queens	N adult workers	N adult males	N queen pupae	N worker pupae	N male pupae	N genotyped male pupae
48	15/05/2017	University of East Anglia, Norwich, Norfolk	52.616029, 1.2348346	2	106	2	21	75	28	24
49*	15/05/2017	University of East Anglia, Norwich, Norfolk	52.616607, 1.2340793	1	63	16	1	73	65	24
50	19/05/2017	Quebec Road, Norwich, Norfolk	52.631360, 1.3142474	1	61	68	1	0	127	24
51	19/05/2017	Peck Close, Norwich, Norfolk	52.640975, 1.2091333	1	93	1	16	0	12	12
52	19/05/2017	Hillside Avenue, Norwich, Norfolk	52.630186, 1.3486132	2	44	22	1	0	75	24
53	20/05/2017	Plash Road, Bedingfield, Suffolk	52.274386, 1.193528	1	99	1	93	4	33	24
54	21/05/2017	Beard Road, Bury St. Edmunds, Suffolk	52.261701, 0.693758	44	47	3	65	1	137	24
55	21/05/2017	Rockingham Road, Bury St. Edmunds, Suffolk	52.237201, 0.720086	11	23	9	8	0	9	9
56	22/05/2017	The Common, Swardeston, Norfolk	52.578986, 1.2429706	2	41	73	10	0	68	24
57	22/05/2017	Back Lane, Rollesby, Norfolk	52.683180, 1.6393086	69	120	36	70	1	62	24
59	25/05/2017	Clarkson Road, Norwich, Norfolk	52.638327, 1.243228	2	83	16	0	0	34	24
60	29/05/2017	Arnott Avenue, Gorleston-on-Sea, Norfolk	52.560394, 1.7262845	17	102	28	0	1	90	24
61	30/05/2017	Brundall Road, Brundall, Norfolk	52.629222, 1.4448819	1	65	1	0	6	12	12
63	31/05/2017	Suton Street, Suton, Norfolk	52.545336, 1.0855051	8	18	3	0	3	7	7
64	31/05/2017	High Bungay Road, Loddon, Norfolk	52.529322, 1.4812202	23	77	25	17	1	55	24
67	12/06/2017	Bunwell Street, Bunwell, Norfolk	52.502842, 1.1133274	40	34	3	32	0	9	9
68	12/06/2017	Church Farm Lane, Scarning, Norfolk	52.680632, 0.93359653	5	6	2	0	0	28	24
181	31/05/2018	Barberry Close, Taverham, Norfolk	52.686703, 1.190012	15	33	7	36	0	20	20
183	12/06/2018	Dog Lane, Horsford, Norfolk	52.695633, 1.231530	1	38	36	0	0	15	15
185	21/06/2018	The Avenues, Norwich, Norfolk	52.626165, 1.263982	6	34	1	7	0	8	8
			**Totals**	252	1187	353	378	165	894	380

Table S2. Collection details of the 2018 field-collected *Bombus hypnorum* queens caught from 7 March to 19 April 2018 at various UK sites and used for laboratory-based colony rearing. Queen ID: identification number of queen. Collection date: date queen collected from field. Collection location: site of queen collection. University of East Anglia Broad refers to the banks of the lake (broad) on the UEA campus. Collection co-ordinates: latitude and longitude of queen collection location.

Queen ID	Collection date (dd/mm/yyyy)	Collection location	Collection co-ordinates (Lat, Long)
74	07/03/2018	Windsor Great Park, Egham, Surrey	51.41746 , -0.60453
75	07/03/2018	Windsor Great Park, Egham, Surrey	51.41746 , -0.60453
76	08/03/2018	Windsor Great Park, Egham, Surrey	51.41820 , -0.60506
77	13/03/2018	Windsor Great Park, Egham, Surrey	51.41820 , -0.60506
78	13/03/2018	Windsor Great Park, Egham, Surrey	51.41820 , -0.60506
79	13/03/2018	Windsor Great Park, Egham, Surrey	51.41746 , -0.60453
80	13/03/2018	Windsor Great Park, Egham, Surrey	51.41766 , -0.60479
81	13/03/2018	Windsor Great Park, Egham, Surrey	51.41820 , -0.60506
82	13/03/2018	Windsor Great Park, Egham, Surrey	51.41849 , -0.60536
83	14/03/2018	Windsor Great Park, Egham, Surrey	51.41766 , -0.60479
84	14/03/2018	Windsor Great Park, Egham, Surrey	51.41746 , -0.60453
85	14/03/2018	Windsor Great Park, Egham, Surrey	51.41849 , -0.60536
86	14/03/2018	Windsor Great Park, Egham, Surrey	51.41746 , -0.60453
87	14/03/2018	Windsor Great Park, Egham, Surrey	51.41820 , -0.60506
88	16/03/2018	Windsor Great Park, Egham, Surrey	51.41820 , -0.60506
89	16/03/2018	Windsor Great Park, Egham, Surrey	51.41820 , -0.60506
90	16/03/2018	Windsor Great Park, Egham, Surrey	51.41820 , -0.60506
91	16/03/2018	Windsor Great Park, Egham, Surrey	51.41820 , -0.60506
92	16/03/2018	Windsor Great Park, Egham, Surrey	51.41849 , -0.60536
93	16/03/2018	Windsor Great Park, Egham, Surrey	51.41806 , -0.60557
94	16/03/2018	Windsor Great Park, Egham, Surrey	51.41820 , -0.60506
95	16/03/2018	Windsor Great Park, Egham, Surrey	51.41849 , -0.60536
96	16/03/2018	Windsor Great Park, Egham, Surrey	51.41820 , -0.60506
97	16/03/2018	Windsor Great Park, Egham, Surrey	51.41820 , -0.60506
98	16/03/2018	Windsor Great Park, Egham, Surrey	51.41806 , -0.60557
99	25/03/2018	Chingford Plain, North Chingford, Greater London	51.63377 , 0.01517
100	25/03/2018	Chingford Plain, North Chingford, Greater London	51.63650 , 0.01380
101	26/03/2018	Chingford Plain, North Chingford, Greater London	51.63377 , 0.01517
102	26/03/2018	Chingford Plain, North Chingford, Greater London	51.63377 , 0.01517
103	26/03/2018	Chingford Plain, North Chingford, Greater London	51.63377 , 0.01517
104	26/03/2018	Chingford Plain, North Chingford, Greater London	51.63377 , 0.01517
105	27/03/2018	Chingford Plain, North Chingford, Greater London	51.63377 , 0.01517
106	23/03/2018	University of East Anglia campus, Norwich, Norfolk	52.6211412, 1.2418573
107	23/03/2018	The Avenues, Norwich, Norfolk	52.625054, 1.2517449
108	26/03/2018	Heigham Park, Norwich, Norfolk	52.6255074, 1.2668717
109	04/04/2018	University of East Anglia Broad, Norwich, Norfolk	52.61853 , 1.23383
110	04/04/2018	University of East Anglia Broad, Norwich, Norfolk	52.61853 , 1.23383
111	04/04/2018	University of East Anglia Broad, Norwich, Norfolk	52.61853 , 1.23383
112	04/04/2018	University of East Anglia Broad, Norwich, Norfolk	52.61853 , 1.23383
113	04/04/2018	University of East Anglia Broad, Norwich, Norfolk	52.61853 , 1.23383
114	04/04/2018	University of East Anglia Broad, Norwich, Norfolk	52.61841 , 1.23418
115	05/04/2018	University of East Anglia Broad, Norwich, Norfolk	52.61853 , 1.23383
116	05/04/2018	University of East Anglia Broad, Norwich, Norfolk	52.61853 , 1.23383
117	05/04/2018	University of East Anglia Broad, Norwich, Norfolk	52.61853 , 1.23383
118	05/04/2018	University of East Anglia Broad, Norwich, Norfolk	52.61853 , 1.23383

119	05/04/2018	University of East Anglia campus, Norwich, Norfolk	52.62107 , 1.24191
120	05/04/2018	University of East Anglia Broad, Norwich, Norfolk	52.61853 , 1.23383
121	05/04/2018	University of East Anglia Broad, Norwich, Norfolk	52.61853 , 1.23383
122	05/04/2018	University of East Anglia Broad, Norwich, Norfolk	52.61853 , 1.23383
123	05/04/2018	University of East Anglia Broad, Norwich, Norfolk	52.61853 , 1.23383
124	05/04/2018	University of East Anglia Broad, Norwich, Norfolk	52.61853 , 1.23383
125	05/04/2018	University of East Anglia Broad, Norwich, Norfolk	52.61853 , 1.23383
126	05/04/2018	University of East Anglia Broad, Norwich, Norfolk	52.61853 , 1.23383
127	05/04/2018	University of East Anglia Broad, Norwich, Norfolk	52.61853 , 1.23383
128	05/04/2018	University of East Anglia Broad, Norwich, Norfolk	52.61853 , 1.23383
129	05/04/2018	University of East Anglia Broad, Norwich, Norfolk	52.61853 , 1.23383
130	05/04/2018	University of East Anglia Broad, Norwich, Norfolk	52.61853 , 1.23383
131	05/04/2018	University of East Anglia Broad, Norwich, Norfolk	52.61853 , 1.23383
132	05/04/2018	University of East Anglia Broad, Norwich, Norfolk	52.61853 , 1.23383
133	05/04/2018	University of East Anglia Broad, Norwich, Norfolk	52.61853 , 1.23383
134	05/04/2018	University of East Anglia Broad, Norwich, Norfolk	52.61853 , 1.23383
135	05/04/2018	Mousehold Heath, Norwich, Norfolk	52.6433580, 1.3203921
136	05/04/2018	Mousehold Heath, Norwich, Norfolk	52.6433580, 1.3203921
137	06/04/2018	University of East Anglia Broad, Norwich, Norfolk	52.61853 , 1.23383
138	06/04/2018	University of East Anglia Broad, Norwich, Norfolk	52.61853 , 1.23383
139	06/04/2018	University of East Anglia Broad, Norwich, Norfolk	52.61853 , 1.23383
140	06/04/2018	University of East Anglia Broad, Norwich, Norfolk	52.61853 , 1.23383
141	06/04/2018	University of East Anglia Broad, Norwich, Norfolk	52.61853 , 1.23383
142	07/04/2018	University of East Anglia Broad, Norwich, Norfolk	52.61853 , 1.23383
143	07/04/2018	University of East Anglia Broad, Norwich, Norfolk	52.61853 , 1.23383
144	07/04/2018	University of East Anglia Broad, Norwich, Norfolk	52.61853 , 1.23383
145	07/04/2018	University of East Anglia Broad, Norwich, Norfolk	52.61853 , 1.23383
146	07/04/2018	University of East Anglia Broad, Norwich, Norfolk	52.61894 , 1.23334
147	07/04/2018	University of East Anglia Broad, Norwich, Norfolk	52.61894 , 1.23334
148	07/04/2018	University of East Anglia Broad, Norwich, Norfolk	52.61853 , 1.23383
149	10/04/2018	St. Catherines Road, Thorpe St. Andrew, Norwich, Norfolk	52.6359227, 1.3536603
150	10/04/2018	St. Catherines Road, Thorpe St. Andrew, Norwich, Norfolk	52.6359227, 1.3536603
151	16/04/2018	University of East Anglia Broad, Norwich, Norfolk	52.61853 , 1.23383
152	16/04/2018	University of East Anglia Broad, Norwich, Norfolk	52.61853 , 1.23383
153	16/04/2018	Bishopgate, Norwich, Norfolk	52.6341603, 1.3047075
154	17/04/2018	University of East Anglia Broad, Norwich, Norfolk	52.61476 , 1.24405
155	17/04/2018	Marston Marshes, Norwich, Norfolk	52.60387 , 1.26708
156	17/04/2018	Marston Marshes, Norwich, Norfolk	52.60387 , 1.26708
157	17/04/2018	Marston Marshes, Norwich, Norfolk	52.60387 , 1.26708
158	17/04/2018	Marston Marshes, Norwich, Norfolk	52.60387 , 1.26708
159	17/04/2018	Marston Marshes, Norwich, Norfolk	52.60387 , 1.26708
160	17/04/2018	Marston Marshes, Norwich, Norfolk	52.60401 , 1.26740
161	17/04/2018	Marston Marshes, Norwich, Norfolk	52.60387 , 1.26708
162	17/04/2018	University of East Anglia Broad, Norwich, Norfolk	52.61091 , 1.24341
163	17/04/2018	University of East Anglia Broad, Norwich, Norfolk	52.61103 , 1.24375
164	17/04/2018	St. Catherines Road, Thorpe St. Andrew, Norwich, Norfolk	52.6357765, 1.3538240

165	17/04/2018	St. Catherines Road, Thorpe St. Andrew, Norwich, Norfolk	52.6361048, 1.3541833
166	17/04/2018	Church Lane, Norwich, Norfolk	52.6046992, 1.2566659
167	18/04/2018	Marston Marshes, Norwich, Norfolk	52.60387 , 1.26708
168	18/04/2018	Marston Marshes, Norwich, Norfolk	52.60387 , 1.26708
169	18/04/2018	Marston Marshes, Norwich, Norfolk	52.60387 , 1.26708
170	18/04/2018	University of East Anglia Broad, Norwich, Norfolk	52.61103 , 1.24375
171	18/04/2018	University of East Anglia Broad, Norwich, Norfolk	52.61091 , 1.24341
172	18/04/2018	University of East Anglia Broad, Norwich, Norfolk	52.61091 , 1.24341
173	18/04/2018	Bishopgate, Norwich, Norfolk	52.6341252, 1.3048479
174	18/04/2018	Bishopgate, Norwich, Norfolk	52.6341252, 1.3048479
175	19/04/2018	Bishopgate, Norwich, Norfolk	52.6342074, 1.3045415
176	19/04/2018	Bishopgate, Norwich, Norfolk	52.6341587, 1.3048070
177	19/04/2018	Bishopgate, Norwich, Norfolk	52.6341292, 1.3047249
178	19/04/2018	Bishopgate, Norwich, Norfolk	52.6341271, 1.3047224
179	19/04/2018	Bishopgate, Norwich, Norfolk	52.6340861, 1.3046055
180	19/04/2018	Bishopgate, Norwich, Norfolk	52.6340800, 1.3045239

Table S3. Colony demographic data for the 37 *Bombus hypnorum* colonies reared from the 2018 field-collected queens. Colony ID: identification number of colony (i.e. same as the Table S2 queen ID for the queen founding a given colony). Asterisk indicates the colony produced diploid males. Population: collection site from which the queen was collected (Table S2). N adult gynes, workers, males: total numbers of adult queens (gynes), workers, and males produced in each colony over the rearing period. N genotyped adult males: number of adult males sampled (in chronological order of eclosion) and genotyped in the current study. N genotyped first-brood males: of all males genotyped, number that were first-brood males (males that eclosed within one week of first worker eclosion or produced by colonies producing no workers). N diploid first-brood males: number of first-brood males genetically confirmed as diploid. N genotyped late males: of all males genotyped, number that were late males (males that eclosed later than one week after first worker eclosion). N diploid late males: number of late males genetically confirmed as diploid. N/A, not applicable, i.e. because no males produced or no males of a given class were produced.

Colony ID	Population	N adult gynes	N adult workers	N adult males	N genotyped adult males	N genotyped first-brood males	N diploid first-brood males	N genotyped late males	N diploid late males
75	Surrey	16	60	209	10	N/A	N/A	10	0
76	Surrey	0	0	3	3	3	0	N/A	N/A
78	Surrey	0	10	62	10	N/A	N/A	10	0
81	Surrey	0	2	0	N/A	N/A	N/A	N/A	N/A
83*	Surrey	0	14	25	24	N/A	N/A	24	4
85	Surrey	0	2	0	N/A	N/A	N/A	N/A	N/A
86	Surrey	0	1	1	1	1	0	N/A	N/A
89	Surrey	0	2	4	4	2	0	2	0
94	Surrey	0	53	186	10	N/A	N/A	10	0
95	Surrey	53	87	83	10	N/A	N/A	10	0
98	Surrey	70	74	150	10	N/A	N/A	10	0
100	Greater London	15	21	8	8	N/A	N/A	8	0
102*	Greater London	0	0	1	1	1	1	N/A	N/A
104	Greater London	0	2	2	2	N/A	N/A	2	0
107	Norwich	22	109	108	10	N/A	N/A	10	0
115	Norwich	8	81	227	10	N/A	N/A	10	0
117	Norwich	0	79	0	N/A	N/A	N/A	N/A	N/A
120	Norwich	0	15	35	24	7	0	17	0
130	Norwich	2	48	17	10	N/A	N/A	10	0
131	Norwich	0	86	130	10	N/A	N/A	10	0
132	Norwich	0	1	0	N/A	N/A	N/A	N/A	N/A
133*	Norwich	0	5	5	5	1	1	4	4
135	Norwich	0	0	2	2	2	0	N/A	N/A
138*	Norwich	0	34	31	24	1	1	23	13
141	Norwich	0	0	3	3	3	0	N/A	N/A
144	Norwich	1	106	114	10	N/A	N/A	10	0
146	Norwich	0	0	2	2	2	0	N/A	N/A
151	Norwich	0	5	6	6	N/A	N/A	6	0

156	Norwich	0	1	0	N/A	N/A	N/A	N/A	N/A
159*	Norwich	0	39	14	14	1	1	13	8
162	Norwich	0	73	0	N/A	N/A	N/A	N/A	N/A
164	Norwich	0	0	1	1	1	0	N/A	N/A
168	Norwich	0	19	0	N/A	N/A	N/A	N/A	N/A
169	Norwich	0	67	0	N/A	N/A	N/A	N/A	N/A
173	Norwich	0	3	0	N/A	N/A	N/A	N/A	N/A
174	Norwich	0	77	7	7	N/A	N/A	7	0
179	Norwich	0	6	1	1	N/A	N/A	1	0
	**Totals**	187	1182	1437	232	25	4	207	29

Table S4. Patterns of male production in colonies reared from the 2018 field-collected *Bombus hypnorum* queens in first-brood male-producing colonies (i.e. those colonies where males eclosed within one week of first worker eclosion or where only males and no workers eclosed). Colony ID: identification number of colony. DMP: whether or not genetically confirmed diploid males were found to have been produced in the colony (Y = found, N = not found). Date of first worker eclosion: date colony's first worker eclosed. Male ID: identification number given to each male in order of eclosion (i.e. male 1 represents the first male to eclose, male 2 represents the second male to eclose, and so on). Male eclosion date: date focal male recorded as eclosing. Days between first worker and focal male eclosion: for focal male, number of days between date of first worker eclosion and male eclosion date. N/A, not applicable. Male ploidy: ploidy of focal male, confirmed by genotyping at up to 14 microsatellite loci.

Colony ID	DMP	Date of first worker eclosion (dd/mm/yyyy)	Male ID	Male eclosion date (dd/mm/yyyy)	Days between first worker and focal male eclosion	Male ploidy
76	N	N/A	1	11/04/2018	N/A	Haploid
			2	16/04/2018	N/A	Haploid
			3	19/04/2018	N/A	Haploid
86	N	12/05/2018	1	12/05/2018	0	Haploid
89	N	30/05/2018	1	01/06/2018	2	Haploid
			2	03/06/2018	4	Haploid
			3	25/06/2018	26	Haploid
			4	28/06/2018	29	Haploid
102	Y	N/A	1	28/04/2018	N/A	Diploid
120	N	26/05/2018	1	30/05/2018	4	Haploid
			2	30/05/2018	4	Haploid
			3	30/05/2018	4	Haploid
			4	31/05/2018	5	Haploid
			5	31/05/2018	5	Haploid
			6	01/06/2018	6	Haploid
			7	01/06/2018	6	Haploid
			8	25/06/2018	30	Haploid
			9	25/06/2018	30	Haploid
			10	25/06/2018	30	Haploid
			11	26/06/2018	31	Haploid
			12	27/06/2018	32	Haploid
			13	27/06/2018	32	Haploid
			14	28/06/2018	33	Haploid
			15	03/07/2018	38	Haploid
			16	06/07/2018	41	Haploid
			17	07/07/2018	42	Haploid
			18	07/07/2018	42	Haploid
			19	10/07/2018	45	Haploid
			20	10/07/2018	45	Haploid
			21	10/07/2018	45	Haploid
			22	10/07/2018	45	Haploid
			23	10/07/2018	45	Haploid
			24	10/07/2018	45	Haploid
133	Y	01/05/2018	1	06/05/2018	5	Diploid
			2	21/05/2018	20	Diploid
			3	22/05/2018	21	Diploid
			4	22/05/2018	21	Diploid
			5	23/05/2018	22	Diploid
135	N	N/A	1	25/05/2018	N/A	Haploid
			2	25/05/2018	N/A	Haploid
138	Y	02/05/2018	1	07/05/2018	5	Diploid
			2	22/05/2018	20	Diploid
			3	28/05/2018	26	Diploid
			4	28/05/2018	26	Diploid

			5	05/06/2018	34	Diploid
			6	05/06/2018	34	Diploid
			7	06/06/2018	35	Diploid
			8	07/06/2018	36	Haploid
			9	10/06/2018	39	Diploid
			10	12/06/2018	41	Diploid
			11	12/06/2018	41	Haploid
			12	13/06/2018	42	Diploid
			13	13/06/2018	42	Haploid
			14	13/06/2018	42	Diploid
			15	13/06/2018	42	Haploid
			16	14/06/2018	43	Haploid
			17	15/06/2018	44	Haploid
			18	16/06/2018	45	Diploid
			19	16/06/2018	45	Haploid
			20	16/06/2018	45	Diploid
			21	18/06/2018	47	Diploid
			22	18/06/2018	47	Haploid
			23	20/06/2018	49	Haploid
			24	21/06/2018	50	Haploid
141	N	N/A	1	11/06/2018	N/A	Haploid
			2	11/06/2018	N/A	Haploid
			3	12/06/2018	N/A	Haploid
146	N	N/A	1	05/06/2018	N/A	Haploid
			2	14/06/2018	N/A	Haploid
159	Y	08/05/2018	1	12/05/2018	4	Diploid
			2	30/05/2018	22	Diploid
			3	01/06/2018	24	Diploid
			4	01/06/2018	24	Diploid
			5	06/06/2018	29	Haploid
			6	07/06/2018	30	Diploid
			7	08/06/2018	31	Diploid
			8	09/06/2018	32	Haploid
			9	09/06/2018	32	Haploid
			10	09/06/2018	32	Haploid
			11	09/06/2018	32	Diploid
			12	10/06/2018	33	Diploid
			13	10/06/2018	33	Diploid
			14	10/06/2018	33	Haploid
164	N	N/A	1		N/A	Haploid

Table S5. Patterns of male production in colonies reared from the 2018 field-collected *Bombus hypnorum* queens in late male producing colonies (i.e. those colonies where males eclosed one week or later after first worker eclosion). Colony ID: identification number of colony. DMP: whether or not genetically confirmed diploid males were found to have been produced in the colony (Y = found, N = not found). Date of first worker eclosion: date colony's first worker eclosed. Male ID: identification number given to each male in order of eclosion (i.e. male 1 represents the first male to eclose, male 2 represents the second male to eclose, and so on). Male eclosion date: date focal male recorded as eclosing. Days between first worker and focal male eclosion: for focal male, number of days between date of first worker eclosion and male eclosion date. Male ploidy: ploidy of each male, confirmed by genotyping at up to 14 microsatellite loci.

Colony ID	DMP	Date of first worker eclosion (dd/mm/yyyy)	Male ID	Male eclosion date (dd/mm/yyyy)	Days between first worker and focal male eclosion	Male ploidy
75	N	03/04/2018	1	12/05/2018	39	Haploid
			2	12/05/2018	39	Haploid
			3	13/05/2018	40	Haploid
			4	13/05/2018	40	Haploid
			5	13/05/2018	40	Haploid
			6	13/05/2018	40	Haploid
			7	14/05/2018	41	Haploid
			8	14/05/2018	41	Haploid
			9	14/05/2018	41	Haploid
			10	14/05/2018	41	Haploid
78	N	11/04/2018	1	18/05/2018	37	Haploid
			2	19/05/2018	38	Haploid
			3	20/05/2018	39	Haploid
			4	24/05/2018	43	Haploid
			5	27/05/2018	46	Haploid
			6	27/05/2018	46	Haploid
			7	27/05/2018	46	Haploid
			8	29/05/2018	48	Haploid
			9	29/05/2018	48	Haploid
			10	30/05/2018	49	Haploid
83	Y	13/04/2018	1	09/05/2018	26	Haploid
			2	09/05/2018	26	Haploid
			3	11/05/2018	28	Diploid
			4	11/05/2018	28	Haploid
			5	12/05/2018	29	Diploid
			6	22/05/2018	39	Diploid
			7	22/05/2018	39	Haploid
			8	25/05/2018	42	Haploid
			9	25/05/2018	42	Haploid
			10	28/05/2018	45	Haploid
			11	28/05/2018	45	Diploid
			12	28/05/2018	45	Haploid
			13	28/05/2018	45	Haploid
			14	28/05/2018	45	Haploid
			15	28/05/2018	45	Haploid
			16	30/05/2018	47	Haploid
			17	01/06/2018	49	Haploid
			18	01/06/2018	49	Haploid
			19	02/06/2018	50	Haploid
			20	03/06/2018	51	Haploid
			21	03/06/2018	51	Haploid
			22	03/06/2018	51	Haploid
			23	03/06/2018	51	Haploid
			24	12/06/2018	60	Haploid

94	N	18/04/2018	1	07/05/2018	19	Haploid
			2	16/05/2018	28	Haploid
			3	17/05/2018	29	Haploid
			4	17/05/2018	29	Haploid
			5	18/05/2018	30	Haploid
			6	18/05/2018	30	Haploid
			7	27/05/2018	39	Haploid
			8	29/05/2018	41	Haploid
			9	29/05/2018	41	Haploid
			10	30/05/2018	42	Haploid
95	N	10/04/2018	1	20/05/2018	40	Haploid
			2	20/05/2018	40	Haploid
			3	20/05/2018	40	Haploid
			4	20/05/2018	40	Haploid
			5	21/05/2018	41	Haploid
			6	21/05/2018	41	Haploid
			7	21/05/2018	41	Haploid
			8	21/05/2018	41	Haploid
			9	22/05/2018	42	Haploid
			10	22/05/2018	42	Haploid
98	N	10/04/2018	1	16/05/2018	36	Haploid
			2	16/05/2018	36	Haploid
			3	16/05/2018	36	Haploid
			4	16/05/2018	36	Haploid
			5	16/05/2018	36	Haploid
			6	16/05/2018	36	Haploid
			7	17/05/2018	37	Haploid
			8	17/05/2018	37	Haploid
			9	17/05/2018	37	Haploid
			10	17/05/2018	37	Haploid
100	N	20/04/2018	1	17/05/2018	27	Haploid
			2	27/05/2018	37	Haploid
			3	27/05/2018	37	Haploid
			4	30/05/2018	40	Haploid
			5	30/05/2018	40	Haploid
			6	01/06/2018	42	Haploid
			7	19/06/2018	60	Haploid
			8	25/06/2018	66	Haploid
104	N	29/04/2018	1	31/05/2018	32	Haploid
			2	11/06/2018	43	Haploid
107	N	27/04/2018	1	25/05/2018	28	Haploid
			2	04/06/2018	38	Haploid
			3	05/06/2018	39	Haploid
			4	06/06/2018	40	Haploid
			5	06/06/2018	40	Haploid
			6	06/06/2018	40	Haploid

			7	06/06/2018	40	Haploid
			8	06/06/2018	40	Haploid
			9	07/06/2018	41	Haploid
			10	09/06/2018	43	Haploid
115	N	28/04/2018	1	30/05/2018	32	Haploid
			2	31/05/2018	33	Haploid
			3	31/05/2018	33	Haploid
			4	31/05/2018	33	Haploid
			5	31/05/2018	33	Haploid
			6	01/06/2018	34	Haploid
			7	01/06/2018	34	Haploid
			8	01/06/2018	34	Haploid
			9	01/06/2018	34	Haploid
			10	01/06/2018	34	Haploid
130	N	02/05/2018	1	02/06/2018	31	Haploid
			2	20/06/2018	49	Haploid
			3	21/06/2018	50	Haploid
			4	21/06/2018	50	Haploid
			5	21/06/2018	50	Haploid
			6	21/06/2018	50	Haploid
			7	22/06/2018	51	Haploid
			8	22/06/2018	51	Haploid
			9	22/06/2018	51	Haploid
			10	22/06/2018	51	Haploid
131	N	27/04/2018	1	30/05/2018	33	Haploid
			2	30/05/2018	33	Haploid
			3	31/05/2018	34	Haploid
			4	31/05/2018	34	Haploid
			5	31/05/2018	34	Haploid
			6	31/05/2018	34	Haploid
			7	31/05/2018	34	Haploid
			8	31/05/2018	34	Haploid
			9	31/05/2018	34	Haploid
			10	31/05/2018	34	Haploid
144	N	29/04/2018	1	02/06/2018	34	Haploid
			2	02/06/2018	34	Haploid
			3	02/06/2018	34	Haploid
			4	02/06/2018	34	Haploid
			5	02/06/2018	34	Haploid
			6	02/06/2018	34	Haploid
			7	02/06/2018	34	Haploid
			8	02/06/2018	34	Haploid
			9	02/06/2018	34	Haploid
			10	03/06/2018	35	Haploid
151	N	12/05/2018	1	31/05/2018	19	Haploid
			2	18/06/2018	37	Haploid

			3	25/06/2018	44	Haploid
			4	25/06/2018	44	Haploid
			5	09/07/2018	58	Haploid
			6	17/07/2018	66	Haploid
174	N	08/05/2018	1	12/06/2018	35	Haploid
			2	14/06/2018	37	Haploid
			3	14/06/2018	37	Haploid
			4	15/06/2018	38	Haploid
			5	15/06/2018	38	Haploid
			6	15/06/2018	38	Haploid
			7	15/06/2018	38	Haploid
179	N	26/05/2018	1	25/06/2018	30	Haploid

Table S6. Genetic diversity data for each of the 13 microsatellite loci at which *Bombus hypnorum* males (pupal or adult) were genotyped and that were used in the analyses (*n* of genotyped males = 612). Locus: the name of each microsatellite locus. N_A: number of unique alleles found at each microsatellite locus. Error rate: the error rate from mistyping at each microsatellite locus. Alleles found at each locus are then numbered 1-8 in base pair size order. Length (bp): the length in base pairs of the allele identified at each microsatellite locus. Freq.: the frequency of the identified alleles at each microsatellite locus across the genotyped males.

Locus	N_A	Error rate	Allele 1		Allele 2		Allele 3		Allele 4		Allele 5		Allele 6		Allele 7		Allele 8	
			Length (bp)	Freq.	Length (bp)	Freq.	Length (bp)	Freq.	Length (bp)	Freq.	Length (bp)	Freq.	Length (bp)	Freq.	Length (bp)	Freq.	Length (bp)	Freq.
B131	5	0	118	0.107	120	0.744	121	0.009	127	0.123	130	0.019						
BL03	6	0.0025	144	0.356	146	0.033	147	0.002	148	0.022	150	0.578	151	0.010				
BT26	6	0	97	0.050	98	0.005	101	0.615	103	0.146	105	0.019	110	0.166				
BTMS0125	8	0.0045	110	0.113	113	0.183	125	0.437	132	0.002	137	0.063	144	0.178	148	0.015	149	0.009
B11	5	0.0045	156	0.003	158	0.260	159	0.066	162	0.551	164	0.179						
B10	6	0.073	191	0.587	192	0.047	193	0.0886	194	0.0646	199	0.0033	200	0.2094				
BTMS0057	5	0	104	0.0914	107	0.1871	109	0.4371	111	0.0403	113	0.2441						
BT05	4	0.005	153	0.0083	156	0.5817	158	0.1142	162	0.2958								
BTMS0056	3	0	254	0.0399	256	0.9235	257	0.0366										
B121	5	0.0255	153	0.1844	159	0.0341	168	0.2676	170	0.4552	208	0.0586						
BTERN02	7	0.0045	157	0.0351	161	0.0017	163	0.3227	165	0.357	167	0.0577	175	0.0084	179	0.2174		
BTMS0083	6	0.0445	277	0.2786	279	0.0111	294	0.0886	302	0.2288	304	0.0203	306	0.3727				
BL08	3	0	145	0.9322	146	0.0018	149	0.066										

Table S7. Ploidy assignments for males sampled from *Bombus hypnorum* colonies based on the number of heterozygous microsatellite loci required to assign a male as diploid. Source of males: set of colonies from which sampled males were genotyped. Colony ID: identification number of colony. ≥ 1 heterozygous loci = diploid: ploidy assignment of males (numbers of haploid or diploid males), under which any male heterozygous at one or more loci was classed as diploid. ≥ 2 heterozygous loci = diploid: ploidy assignment of males (numbers of haploid or diploid males), under which any male heterozygous at two or more loci was classed as diploid. ≥ 3 heterozygous loci = diploid: ploidy assignment of males (numbers of haploid or diploid males), under which any male heterozygous at three or more loci was classed as diploid. Bold colony ID represents the single colony (colony 49) in which the number of diploid males changed depending on the number of heterozygous microsatellite loci required to assign a male as diploid.

Source of males	Colony ID	≥ 1 heterozygous loci = diploid		≥ 2 heterozygous loci = diploid		≥ 3 heterozygous loci = diploid	
		Haploid males (N)	Diploid males (N)	Haploid males (N)	Diploid males (N)	Haploid males (N)	Diploid males (N)
Field-collected colonies (male pupae)	48	24	0	24	0	24	0
	**49**	7	17	7	17	8	16
	50	24	0	24	0	24	0
	51	12	0	12	0	12	0
	52	24	0	24	0	24	0
	53	24	0	24	0	24	0
	54	24	0	24	0	24	0
	55	9	0	9	0	9	0
	56	24	0	24	0	24	0
	57	24	0	24	0	24	0
	59	24	0	24	0	24	0
	60	24	0	24	0	24	0
	61	12	0	12	0	12	0
	63	7	0	7	0	7	0
	64	24	0	24	0	24	0
	67	9	0	9	0	9	0
	68	24	0	24	0	24	0
	181	20	0	20	0	20	0
	183	15	0	15	0	15	0
	185	8	0	8	0	8	0
	**Total**	363	17	363	17	364	16
Field-collected queen reared (first-brood male-producing)	76	3	0	3	0	3	0
	86	1	0	1	0	1	0
	89	4	0	4	0	4	0
	102	0	1	0	1	0	1
	120	24	0	24	0	24	0
	133	0	5	0	5	0	5
	135	2	0	2	0	2	0
	138	10	14	10	14	10	14
	141	3	0	3	0	3	0
	146	2	0	2	0	2	0
	159	5	9	5	9	5	9
	164	1	0	1	0	1	0
	**Total**	55	29	55	29	55	29
Field-collected queen reared (late male-producing)	75	10	0	10	0	10	0
	78	10	0	10	0	10	0
	83	20	4	20	4	20	4
	94	10	0	10	0	10	0
	95	10	0	10	0	10	0
	98	10	0	10	0	10	0
	100	8	0	8	0	8	0
	104	2	0	2	0	2	0
	107	10	0	10	0	10	0
	115	10	0	10	0	10	0
	130	10	0	10	0	10	0
	131	10	0	10	0	10	0
	144	10	0	10	0	10	0
	151	6	0	6	0	6	0
	174	7	0	7	0	7	0
	179	1	0	1	0	1	0
	**Total**	144	4	144	4	144	4

Table S8. Expected heterozygosity (H_e) values for the UK *Bombus hypnorum* study population and populations of common, established, and widespread ('Common') or scarce, range-restricted, and/or declining ('Scarce') *Bombus* species in the UK and Belgium. N, number of workers sampled; where original studies gave multiple values (i.e. from multiple populations), mean H_e and the range of worker numbers sampled are shown. Dash (-), comparable microsatellite data not available for a given species. Belgium was used for comparison with the UK because appropriate data were available and it represents a continental European population with *Bombus* species population statuses comparable to those in the UK.

Population status	Species	UK H_e	UK N	Belgium H_e	Belgium N	Source(s)
Common	*B. hypnorum*	0.51	645	0.38	8-10	[22]
Common	*B. hortorum*	0.84	88	0.57	17-25	[10,22,23]
Common	*B. lapidarius*	0.74	88	0.74	19-24	[10,22,23]
Common	*B. pascuorum*	0.56	32-237	0.44	19-26	[10,16,22,23]
Common	*B. pratorum*	-	-	0.61	19-22	[23]
Common	*B. terrestris*	0.81	88	-	-	[10]
Scarce	*B. distinguendus*	0.39	15-370	-	-	[17]
Scarce	*B. humilis*	-	-	0.35	8	[23]
Scarce	*B. muscorum*	0.45	23-119	-	-	[15]
Scarce	*B. ruderarius*	-	-	0.31	6-11	[22,23]
Scarce	*B. ruderatus*	0.75	88	-	-	[10]
Scarce	*B. soroeensis*	-	-	0.43	9	[22,23]
Scarce	*B. sylvarum*	0.41	18-40	0.34	13-15	[16,22,23]
